# Eyes in the sky: Drone monitoring of the largest gharial and mugger populations in the East Rapti River, Chitwan National Park

**DOI:** 10.1371/journal.pone.0330350

**Published:** 2025-08-21

**Authors:** Gokarna Jung Thapa, Kanchan Thapa, Shashank Poudel, Dil Bahadur Purja Pun, Sujita Shrestha, Prem Poudel, Hari Bhadra Acharya, Bal Kumar Lamsal, Rajesh Sada, Serge A. Wich

**Affiliations:** 1 WWF Nepal, Kathmandu, Nepal; 2 Department of National Parks and Wildlife Conservation, Kathmandu, Nepal; 3 Terai Arc Landscape Program, Kohalpur, Banke, Nepal; 4 Geovation Nepal, Kathmandu, Nepal; 5 School of Biological and Environmental Sciences, Liverpool John Moores University, Liverpool, United Kingdom; Chitkara University, INDIA

## Abstract

Drone-based aerial monitoring can play a pivotal role in scaling up efforts to monitor species at risk. In this study, we assessed the population size, occupancy, and spatial interactions of gharials and muggers in the Eastern Rapti River and its tributaries within Chitwan National Park, complying with national regulations. Using a Wingtra Tail-Sitter Vertical Take-Off and Landing fixed-wing drone, we surveyed a 73-km river stretch during the species’ basking period. The drone captured 24,129 photographs across 27 flight missions, covering 702.66 km and 44.68 km², of which 153 contained dorsal images of gharials (77) and muggers (76). An experienced image analyst identified and counted 323 crocodiles (205 gharials and 118 muggers) from the images. The encounter rates were 14.33 gharials and 9.95 muggers detections per 1 hour of drone flight time. To measure habitat-use through an occupancy framework, we divided the 73-km river stretch into 809 grid cells of 0.04 km² each. The site-level probabilities of habitat-use were 0.47 for gharials and 0.24 for muggers. As anticipated, both species co-occurred spatially along the Eastern Rapti River during the winter season, with a spatial interaction factor (SIF) of 1.94. This study demonstrates the effectiveness of drones in collecting high-resolution ecological data—both spatial and temporal—for assessing population parameters and monitoring threatened crocodile species at scale. Drones offer a cost-effective and less labor-intensive (~US$ 0.61 per km) alternative to traditional ground-based surveys (~US$ 21 per km). Integrating machine learning with drone surveys for automated image analyses has significant potential to further reduce costs and increase efficiency and could strengthen conservation efforts across South Asian River system.

## Introduction

Wildlife managers and researchers have adopted various methods to enhance research and monitoring systems, enabling them to gather critical information on the ground [1]. Rapid technological advancements are revolutionizing wildlife monitoring techniques [2,3]. Tools such as camera traps and drones have recently gained popularity and leverage advancing technologies in wildlife management [4,5]. While camera traps are widely used, drones are a relatively new addition to wildlife studies and have primarily been applied for species detection [5], and estimating wildlife density and abundance which have increased in recent times ([6] in Delacour’s langur). In recent years, drones have been employed across various civilian disciplines, including agriculture [7], forestry [8], biodiversity monitoring [9], and wildlife research [10–12]. The integration of drones with high resolution spatial and temporal data in monitoring threatened species or at-risk populations is not only valuable but also increasingly essential [1,12,13].

The gharial (*Gavialis gangeticus*) and mugger (*Crocodylus palustris*) are two threatened ectothermic crocodilians categorized as *Critically Endangered* (gharial) and *vulnerable* (mugger) under IUCN Red List [14,15]. Gharials are primarily restricted to flowing freshwater systems, whereas muggers are generalists, inhabiting a wider range of habitats including rivers, lakes, and marshes [16,17]. Monitoring threatened species and their habitats, including those at risk, is essential for tracking population trends, determining the urgency of conservation actions, and evaluating the effectiveness of interventions [18]. In Nepal, studies on crocodilians have increased recently [19–25] and rely mainly on traditional ground-based surveys, that employs dugout canoe or boats, for data collections. Ground-based population surveys often underestimate counts and are often affected by disturbances before detection [26]. However, the application of drones in crocodilian and/or most of wildlife research remains limited in Nepal. Thapa *et al.,* [26] pioneered the use of drones for freshwater species monitoring with research focusing on crocodiles in Bardia National Park. The monitoring framework developed by Thapa *et al.,* [26] provided a foundation for scaling up crocodilian monitoring to larger areas. This study is the first to use drones to monitor the largest populations of gharials and muggers in Nepal’s River systems [22,25,27]. Using higher-precision drone platforms-relating to choice of equipment, flight approach speed and altitude flight, choice of species and appropriate seasons- can minimize disturbance and nuisance to wildlife [28,29]. This paper demonstrates a low-disturbance approach to advancing crocodilian conservation and research.

Nepal government periodically releases gharial from its breeding facilities into the East Rapti River to supplement species stock in the wild [25,27]. From 1981 to 2025, a total of 1,305 gharials has been released from the captive breeding center into East Rapti River in Chitwan National Park (CNP) [30]. Limited ecological data and monitoring tools often hinder effective tracking of population performance and survival tendencies in the wild after release. Many ground-based surveys reported total counts and/or reported activity index [20,25,31] which are often underestimated [26]. In recent times, ground-based surveys with replication are used to estimate gharial population size [22]. Given the pervasive threats from habitat loss and overexploitation of their prey, such as fish [27], the lack of timely and robust ecological data on the distribution and abundance of gharials and muggers is a significant concern for conservationists,. Given the rapid advancement in technology, drones have emerged as a cost-effective and reliable tool for monitoring different species, crocodilian populations, minimizing observer bias [26,32,33]. This study evaluates the effectiveness of drones compared to traditional ground-based surveys for monitoring gharials and muggers. It also provides essential ecological insights into habitat occupancy and population abundance, and their interactions supporting the long-term conservation of these species in the East Rapti River system of CNP.

Gharials and muggers are considered as a sympatric species in South Asia [34] including Nepal. We used detection and non-detection data of species from dorsal drone images within an occupancy modeling framework [35,36] while accounting for imperfect detection to evaluate the distribution, habitat use, and co-occurrence of gharials and muggers. We hypothesized that the East Rapti River (Here after referred as “Rapti”) supports the largest population of gharials and muggers, which can be visually identified as dorsal images under drone platform, as corroborated by ground-based survey results [22,25]. Additionally, we hypothesized that gharials and muggers co-occur along Rapti River more frequently than expected by random chance, based on visual identification from drone imagery. To collect this data, we deployed a tail-sitting fixed-wing vertical take-off and land (VTOL) drone, capturing detection and non-detection of our study species in the drone images along the Rapti River and its tributaries in CNP.

## Materials and methods

### Ethics statement

The study involved data collected from aerial monitoring using drones. The drone flights were conducted with due permission from the Government of Nepal-Ministry of Home Affairs (letter reference no: 651, dated 8^th^ February 2024), and Department of National Parks and Wildlife Conservation (letter reference no: 1084, dated 20^th^ November 2023). Animal care and use committee approval was not required as the survey was based on the non-invasive techniques that involves taking photographs from the sky using the drones.

### Study area

**Fig 1:**
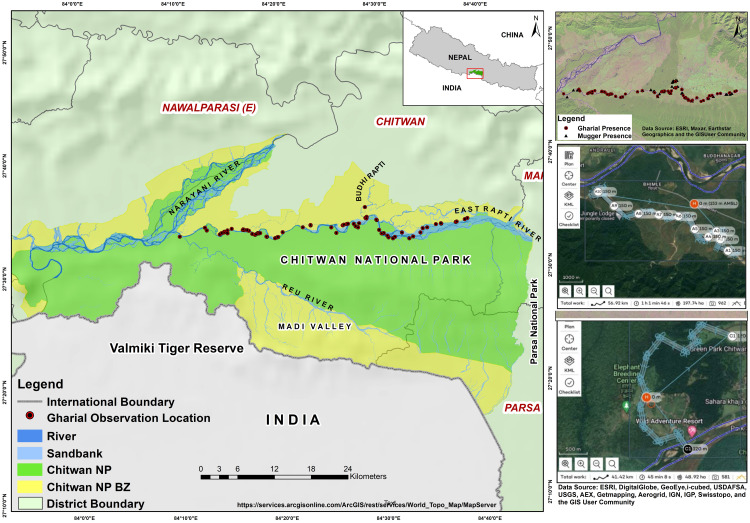
Study area showing the East Rapti River, Reu River, and Budhi Rapti River as freshwater habitats of gharial and mugger in Chitwan National Park. The flight path of drone belongs to one of the pre-designed mission plans [26,27] across the East Rapti River. The mission was designed in *Mission planner* following software manuals. Each light blue line represents coordinates in the respective mission plan of the drone. Map created using ArcGIS. Sources: Esri, DigitalGlobe, GeoEye, i-cubed, USDA FSA, USGS, AEX, Getmapping, Aerogrid, IGN, IGP, swisstopo, and the GIS User Community (https://services.arcgisonline.com/ArcGIS/rest/services/World_Topo_Map/MapServer).

This study was carried out in CNP (27°30′N, 84°20′E) located in south-central Nepal. The Park covers an area of 952.63 km^2^ in the sub-tropical lowland forest of the inner Terai. Three major rivers—Narayani, Rapti, and Reu—flow through CNP, supporting the country’s largest populations of gharials and muggers [25,27]. Due to logistical constraints, we could not survey the Narayani River. As historical records suggests that Rapti River forming the northernmost boundary of the park has been the stronghold for Gharial populations as compared to the Narayani and Reu River, this study focused on surveying the Rapti River. In addition, some lower sections of Reu River was also surveyed in its confluence with Rapti River. Therefore, we selected sampling stretches in the Rapti River and the downstream section of the Reu, totaling 73 km (44.68 km², Fig 1). These include 60 km of the Rapti River, 9 km of the Reu River, and 4 km of the Budhi Rapti River (a tributary of the Rapti). These stretches provide an extensive ribbon of aquatic habitat. The gharial, mugger, and smooth-coated otter (*Lutrogale perspicillata*) are the top freshwater predators found in CNP. A total of 111 fish species have been identified along the Narayani and Rapti River system which provides a source of protein for both gharial and mugger population [37]. The floodplain contains deciduous riverine forests. Early successional stands feature dominant tree species such as *Acacia catechu*, *Dalbergia sissoo*, *Bombax ceiba*, and *Trewia nudiflora*. Late-successional stands include remnants of *Bombax* and *Trewia*, which co-dominate with evergreen species like *Persea* spp., *Syzygium* spp., *Mallotus philippinensis*, *Dysoxylum* spp., and *Ficus racemosa* [38]. The river stretches are an ecological lifeline to majority of species including large mammals such as Asian elephant (*Elephas maximus*), greater one-horned rhinoceros (*Rhinoceros unicornis*), and tiger (*Panthera tigris tigris*). The major habitat types in CNP and surrounding areas includes grassland (176 km^2^), forest (2,957 km^2^), and settlement and agricultural areas (1,312 km^2^). CNP has three distinct seasons: winter, summer, and the monsoon season [39]. The mean annual rainfall is 2,437 mm, with a highly skewed rainfall pattern, and highest rainfall can be observed during the months of July-September [40]. Average ambient temperature varies from a minimum of 11^◦^C in winter to a maximum of 38^◦^C in summer season. More details on the CNP are presented elsewhere [39–41].

### Drone census methodology

We adapted the census methodology from a similar ecological study along the Babai River in Bardia National Park, in western Nepal [26]. A Tail sitter VTOL fixed-wing drone was used to capture images of gharials and muggers along the riverbanks, following pre-programmed aerial routes. The drone’s flight altitude was restricted to 150 meters in compliance with local civil aviation regulations which also ensured minimal disturbance to the species under study [42]. We also recorded any behavioral response from gharial and muggers as an indication of drone disturbance by giving a score of “0” if species do not move from their initial position and a score of “1” if the species move into the water/dive when each is basking or swimming on the surface. We selected the Wingtra One Gen II fixed-wing drone (Wingtra) for the survey due to its availability in the country and its long-range VTOL capability, allowing efficient coverage of large areas with advanced technology. Wingtra One Gen II is equipped with telemetry connection and remote operation (2.4–2.48 GHz) along the range of bi-directional 10 km in direct line of sight. We used Wingtra Pilot (V2.14.0) application for the flight planning and execution. Communication between the drone and ground station (tablet) was enabled through Wingtra One Telemetry 2.4. This drone, with a wingspan of 125 cm and weighing approximately 3.7 kg (with batteries), had a flight duration of around ~ 59 minutes with the speed of 16m/s, making it ideal for its portability and low weight.

The survey required high-resolution cameras capable of capturing images from a 150 m altitude while remaining lightweight and aerodynamically efficient. We used Sony RX1RII camera (Sony Inc.) with a pay load of 590 grams, mounted on the drone platform, to capture photographs in JPG format regularly at 9.6 m intervals at resolution of 42.4 mega pixel with a focal length of 35 mm and an ISO setting of 100.

At an altitude of 150 meters, the camera captures a ground area of approximately 115 meters wide by 130 meters length per image. In post-processing, images from both sides were combined, expanding the total survey coverage to approximately 694.2 km, with some areas requiring three flight paths due to the river’s width.

Season and timing were carefully chosen for optimal conditions. The field survey was conducted in January and February 2024, a period of low turbidity in the Rapti River and its tributaries, allowing sufficient water clarity for potential identification of crocodilians swimming near the surface (~1 m depth). Photographs were taken in the morning and afternoon (11:00 AM – 6:00 PM) to coincide with the crocodiles’ typical basking times [25,42], including those floating along the river in winter, while also avoiding morning haziness when flying at 150 m.

All flights were programmed using Mission Planner software (i.e., Wingtra hub), each mission involving a hand launch and automated landing. The 27 missions conducted (Table 1) adhered to local regulations and collectively covered a 73 km stretch of floodplain habitat along the Rapti River and its tributaries. All flights were piloted by the lead author GJT and co-author BKL.

**Table 1:** Search effort, survey timing, number of photos captured by the drone, and gharial/mugger count in each of the 27 pre-designed missions in the East Rapti River, Chitwan National Park.

Mission Plan	Search Effort				# of total captured photos	# of gharial count	# of mugger count	Gharial OD	Mugger OD	Gharial RAI	Mugger RAI
	Survey Timing	Distance Covered (in km)	Flight Time (in min)	# surface area covered (in km^2^)							
1	11:00 -12:00	35.2	41	3.49	1,589	2	0	0.57	0.00	2.93	0.00
2	12:00 -13:00	26.6	31	1.53	690	0	9	0.00	5.87	0.00	17.42
3	14:00 -15:00	28.3	33	3.33	1,529	3	1	0.90	0.30	5.45	1.82
4	15:00 -16:00	35.2	41	1.94	850	1	13	0.52	6.71	1.46	19.02
5	16:00 -17:00	24.0	28	1.04	512	8	5	7.72	4.83	17.14	10.71
6	11:00 -12:00	30.0	35	2.47	1,304	9	1	3.64	0.40	15.43	1.71
7	12:00 -13:00	30.0	35	1.66	818	10	9	6.04	5.43	17.14	15.43
8	14:00 -15:00	33.5	39	1.92	929	22	10	11.47	5.22	33.85	15.38
9	14:00 -15:00	12.9	15	1.29	631	0	1	0.00	0.78	0.00	4.00
10	15:00 -16:00	35.2	41	2.33	1,180	10	8	4.30	3.44	14.63	11.71
11	11:00 -12:00	8.6	10	1.01	1,081	2	2	1.98	1.98	12.00	12.00
12	11:00 -12:00	15.4	18	0.62	308	10	8	16.05	12.84	33.33	26.67
13	12:00 -13:00	33.5	39	1.78	857	3	0	1.68	0.00	4.62	0.00
14	14:00 -15:00	12.0	14	0.45	784	5	2	11.23	4.49	21.43	8.57
15	15:00 -16:00	29.2	34	1.16	570	32	9	27.50	7.73	56.47	15.88
16	15:00 -16:00	24.0	28	0.89	440	22	4	24.60	4.47	47.14	8.57
17	11:00 -12:00	36.0	42	4.51	2,102	26	0	5.77	0.00	37.14	0.00
18	12:00 -13:00	34.3	40	3.87	1,778	20	0	5.17	0.00	30.00	0.00
19	14:00 -15:00	27.5	32	1.33	1,675	2	0	1.51	0.00	3.75	0.00
20	15:00 -16:00	21.5	25	1.19	579	0	2	0.00	1.68	0.00	4.80
21	16:00 -17:00	12.0	14	0.44	458	1	1	2.29	2.29	4.29	4.29
22	16:00 -17:00	18.0	21	0.64	293	3	1	4.70	1.57	8.57	2.86
23	17:00 -18:00	20.6	24	0.92	423	13	4	14.14	4.35	32.50	10.00
24	12:00 -13:00	33.5	39	1.41	717	0	0	0	0	0.00	0.00
25	13:00 -14:00	21.5	25	1.98	962	0	0	0	0	0.00	0.00
26	16:00 -17:00	21.5	25	1.00	489	0	0	0	0	0.00	0.00
27	12:00 -13:00	32.6	29	0.49	581	1	28	2.04	57.23	2.07	57.93
**Total**		**694.2**	**798**	**44.68**	**24,129**	**205**	**118**	**5.07**	**4.87**	**14.86**	**9.21**

OD: Observed Density; RAI: Relative Abundance Index.

### Drone flight calibration

To ensure accurate geospatial mapping for the gharial survey along the Babai River, the Wingtra One VTOL drone was calibrated using a multi-step process tailored to local coordinate systems. Pre-flight, the drone’s built-in protocols automatically adjusted onboard sensors (e.g., IMU, compass) and camera parameters (e.g., focus, exposure) to optimize data collection.

To enhance positional accuracy, we conducted local calibration using a GPS rover paired with a GNSS (Global Navigation Satellite System) base station receiving both L1 and L2 signals. During the flight, the drone recorded raw GNSS data, while the base station logged positional data. Post-Processing Kinematics (PPK) techniques were then applied to correct the raw data using the base station logs. A global coordinate projection file facilitated transformation of PPK-corrected data into local grid system.

To validate spatial accuracy, we deployed a set of ground control points (GCPs) evenly distributed across the survey area. These GCPs served as reference points for validating the transformed coordinates and assessing the precision of the PPK-derived outputs. Incorporating GCPs into the workflow ensured robust alignment with the local coordinate reference framework.

### Post-production for photo stitching

We used Wingtra Hub to stitch together drone images taken during each mission, resulting in one combined image per mission. We used Pix 4D mapper (www.pix4d.com, Switzerland) as post image processing to generate orthomosaic images of the survey areas. Pix4D is a photogrammetry software that digitizes reality and measures data from images captured by planes, drones, phones, and other devices. The photos were stitched based on their sequential numbering and time stamp, yielding a total of 27 geo-reference orthomosaic images.

### Image geo-rectification

To geo-rectify all the images produced based on the mission plan, ~ 500 GCPs were extracted per image in Pix4D using an algorithm called SIFT. After the extraction, GCPs were used as a key point for matching process for the image geo-rectification in the Pix4D mapper.

### Crocodilian counting approach

For crocodilian census, three image analysts, trained in distinguishing gharial and mugger morphologies, independently reviewed dorsal photographs from 27 drone survey missions. Each analyst counted individual gharials and muggers, with the final count determined through expert arbitration to reach consensus among the three [26]. Photographs with identification discrepancies were excluded from the final dataset. Crocodilian identification of drone images relied on visually assessing morphological characteristics [43]. At an altitude of 150 meters, image resolution did not allow us to distinguish between sexes or age classes, so only the total number of individuals was recorded.

**Fig 2:**
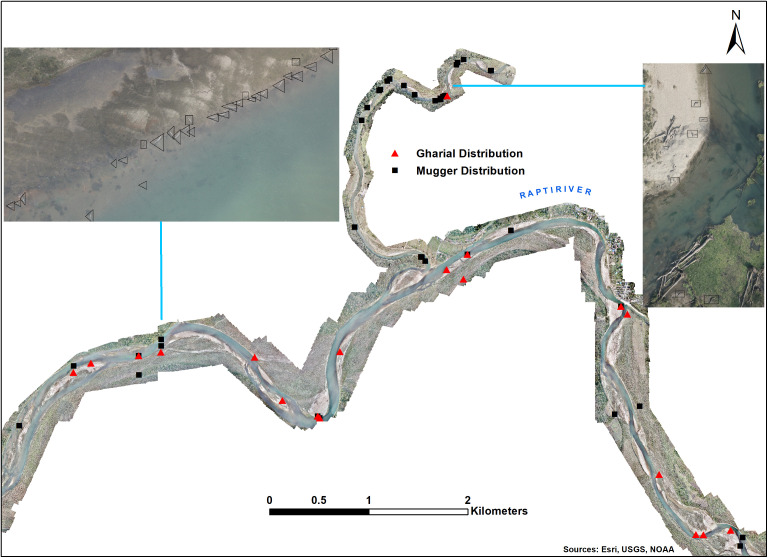
Stitched photograph segments from Mission 15 (29.2 km, 34 min) and Mission 27 (32.6 km, 29 min) showing gharials (▲) and muggers (■) recorded along the study area in Chitwan National Park. The inset illustrates the differentiation between gharials (triangular box) and muggers (rectangular box) based on their physical appearance as observed in drone images and verified by three expert volunteers. Map created using ArcGIS. Sources: Esri, USGS, NOAA (https://services.arcgisonline.com/ArcGIS/rest/services/World_Topo_Map/MapServer).

Two approaches were used for identifying crocodilians in the images [26]. First, each analyst looked for visible clusters and examined the snout shape and length to differentiate between gharials and muggers [43]. Gharials have a long, slender snout, while muggers have a shorter one (Fig 2). Second, consensus-identified samples were further screened using CountThings software (Dynamic Venture, Inc.), which verified clusters identified by the analysts. The software detected and highlighted each object/cluster, which the analysts then manually labeled as either gharial or mugger. We separated all images containing objects (gharial and mugger) from each mission plan and obtained the spatial coordinates of these objects. This approach eliminates the possibility of counting the same objects in two consecutive images. Furthermore, the present study (as detailed in the results section) did not detect any movement of gharials between consecutive mission plans.

### Analytical method

#### Relative abundance index and observed density.

We defined a ‘mission’ as the basic sampling unit. For each mission, we summarized the counts of individuals identified in drone photographs and reported them as drone-derived counts. To maintain consistency, we standardized all drone flights in terms of speed, altitude, and timing. We calculated a simple relative abundance index, defined as the number of drone-derived counts per hour of flight [26,44]. Additionally, we calculated observed density by expressing the number of drone-derived counts per square kilometer of surface area [26]. The total surface area covered totaled 44.68 km² (Table 1).

#### Crocodilian probability of habitat use intensity and spatial co-occurrence.

We employed occupancy modeling techniques using drone derived images to derive stronger inference by decomposing true absence from non-detection within a probabilistic framework [35]. We estimated the grid-level probability of gharial and mugger occupancy (200 m by 200 m grids, or 0.04 km²), which represents the intensity of habitat use along the Rapti River and its tributaries. For crocodilians with larger home ranges (5–25 km^2^, in Chambal River in India [45]) than our 0.04 km^2^ grid cells, occupancy models can be used to describe habitat-use at finer spatial scales rather than true occupancy (e.g., within home range habitat use, see tigers: [46] and elephants: [47]). Gharials and muggers exhibit relatively lower movement levels during winter compared to summer and monsoon seasons [22,45]. Given the drone’s flight speed of 16 m/sec, we assume that the animal did not move significantly between grids covered by adjacent flight paths. We retrieved all detection data from the total sample area of 44.68 km², identified as potential crocodilian habitat. Detections beyond this boundary were excluded from the analysis. The potential habitat within the study area includes seven types of riverbanks [24], running water, and floodplain grasslands. We extracted all usable habitat from the orthomosaic map to model species occupancy (habitat use) and detectability.

We selected a 0.04 km² grid cell as the basic sampling unit, providing sufficient spatial samples to adequately parameterize occupancy analysis and minimizing autocorrelation between consecutive grids caused by smaller grid sizes [48]. We surveyed a 73-km (covering 44.68 km^2^) stretch of the river, dividing it into 809 grid cells and arranged it in a checkerboard pattern to assess habitat use intensity along the Rapti River and its tributaries within Chitwan National Park. Each grid cell was sampled using transect flight paths that covered potential crocodile habitats, including the river, riverbanks, and floodplains. Mission plans were overlaid on the river stretches, and the number of flight paths covering each grid cell was summarized. Drone survey made repeated visits to each grid- with each visit as spatial replicate- within single primary drone survey. Each grid cell was surveyed by 1–5 straight flight paths, with each 200m flight path within a grid considered as spatial replicate. This provides opportunities to develop encounter history for estimating two probabilities: occupancy and detection. For each flight path, we recorded the presence or absence of gharials and muggers from the final orthorectified image, mapped their locations to the corresponding grid cells, and developed detection histories for each species individually or combined across all 0.04 km² grid cells.

We used single-species, single-season occupancy models [49] in Program PRESENCE [50] to assess site use for each species individually and combined. Our primary parameters of interest were probabilities of occupancy and detection. To estimate these, we applied a standard occupancy model (Ψ (.)) and survey specific detectability model (*p* (survey)) within the single species-species framework. Models were evaluated based on Akaike information criteria (AIC) [51]. Because we aimed to estimate the overall proportion of occupied cells during the drone survey, and surveyed all cells within the area of interest, we used conditional occupancy probabilities. These probabilities are conditional upon the actual observations at a particular cell [35]. We further investigated spatial overlap using two-species co-occurrence models [52] for each pair-wise combination of crocodilians with adequate detection data. We considered two species to occur together less often than random (potential avoidance), when φ, the species interaction factor (SIF), was < 1, and to occur together more often than random when φ > 1. Two species were considered spatially independent if φ = 1 or the standard errors overlapped 1.0. Following MacKenzie et al. [52], we developed two models (one with φ estimated and one with φ set = 1) and formally compared fit based on AIC values. We considered models competing if ΔAIC < 2 [51] in both the cases. We summarized the SIF and derived parameter, i.e., probability of habitat uses by both species (gharial and mugger) from the top model.

Lastly, we compared the investment for conducting the drone-based and traditional boat-based surveys to measures their cost effectiveness [53]. We evaluated search effort in terms of US$ per km for each method by measuring financial costs incurred in the field and laboratory. Since we did not conduct a traditional boat survey, we relied on the field protocol from Yadav *et al*., [22] for comparison.

## Result

We flew the drone at a speed of 16 m/s across a total of 27 pre-planned missions, dedicating a total search effort of 13.3 hours (mean flight time: 29.56 minutes, SD: 9.62 minutes). The flights covered a cumulative distance of 694.2 km (mean distance: 25.6 km, SD: 8.4 km) while mapping river surface, riverbank and floodplain habitats (Table 1). During these missions, the drone captured 24,129 photographs, of which we selected approximately 54% (13,171 photographs) for stitching to produce ortho rectified image, covering an effective surface area of 44.68 km² (mean: 1.6 km², SD: 1.1 km²). We carefully examined all the photographs for dorsal images of gharials and/or muggers. Only 153 photographs (about 1.1% of the stitched images) in the final image revealed the presence of these species in the river, on the riverbanks, or within the floodplain habitat. The drone captured images at a resolution of 2 cm per pixel. 27 pre-planned mission flights overlaps with basking period between 11:00 to 18:00 (Table 1). 60% of gharials were counted between 14:00 to 18:00 while for mugger 52% were counted between 14:00 and 18:00 .

Three image analysts independently examined each of the stitched photographs from the 27 missions (Fig 2). They identified a total of 323 crocodiles through consensus: 205 gharials (ranging from 1 to 26 individuals per orthorectified image) and 118 muggers (ranging from 1 to 28 individuals per orthorectified image) in Rapti River. No crocodiles were recorded in the sampled Reu River. We recorded 72 gharials (~35%) and 20 muggers (~17%) floating in the river. These crocodiles were distributed both as clusters and solitary individuals along the Rapti River. Drone surveys identified 30 gharial groups and 29 mugger groups, each with more than two individuals. The largest groups included 26 gharials in the Rapti River and 9 muggers in the Budhi Rapti River. Drone flights at an altitude of 150 m did not alter species positions (100% − 0 score), suggesting that neither species interacted with the flight paths in a way that affected detection capability and species count.

**Fig 3:**
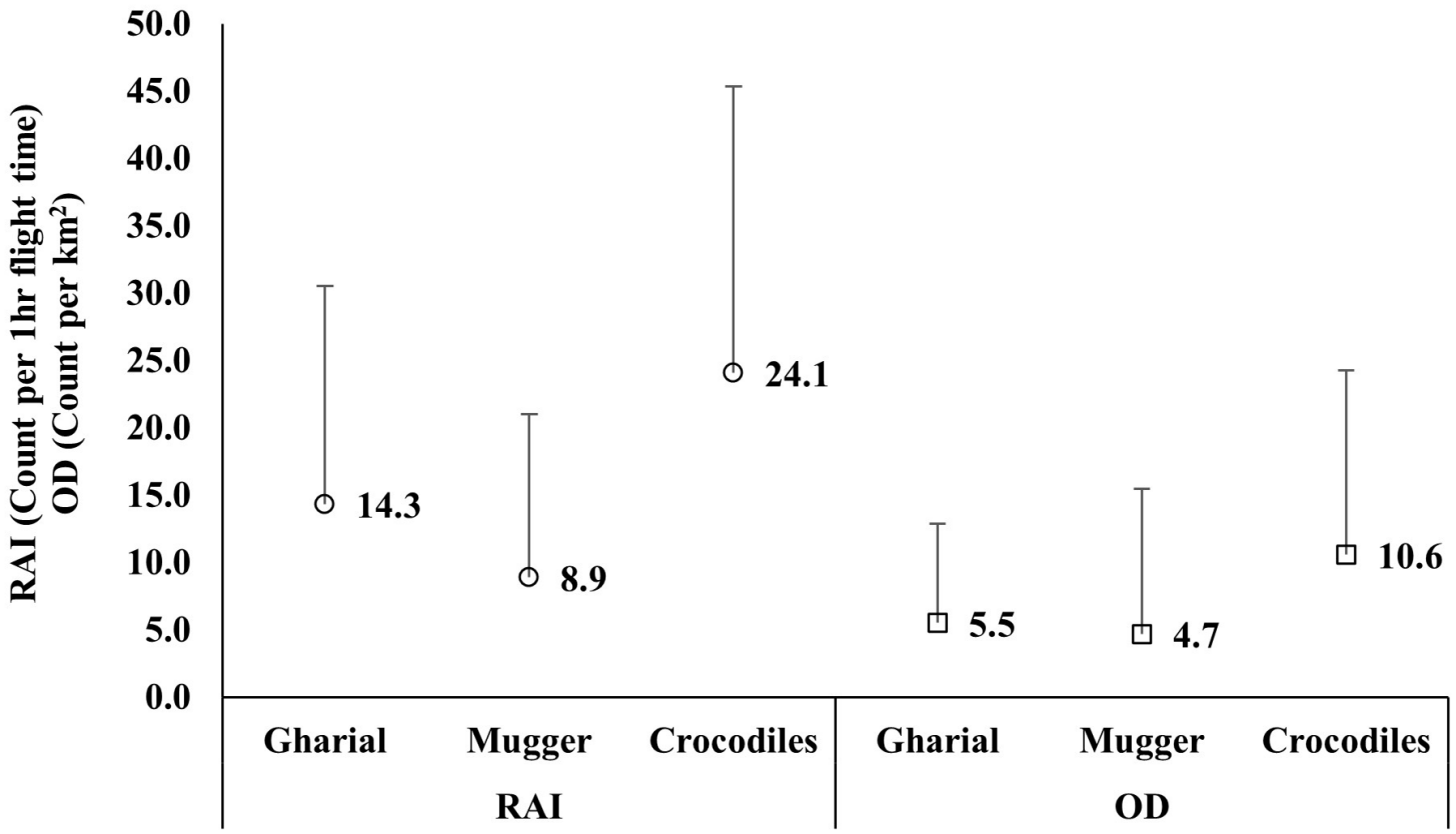
Relative Abundance Index (RAI; °) and Observed Density (OD; ⸋) of gharials, muggers, and crocodiles (combined gharials and muggers) across the study areas in Chitwan National Park. The error bar represents only the upper standard deviation.

We calculated the encounter rate (no of detections per 1 hour of drone flight time) as 14.33 (SD: 16.1) for gharials and 9.95 (SD: 12.41) for muggers (Table 1, Fig 3). The observed density per km² was 5.5 (SD: 7.36) for gharials and 4.7 (SD: 10.8) for muggers (Fig 3), although both species exhibited high standard deviations.

We estimated species occurrence using a single-species, single-season occupancy model that accounted for imperfect detection. We tested two parameterizations: one with a null model for occupancy and survey-specific detection probability, and another with null models for both occupancy and detection. The model with survey-specific detection probability performed best for the species (gharial, mugger, and combined; Table 2).

**Table 2:** Single species single season occupancy model [49] used to estimate probability of habitat use for species (gharial and mugger) at the 0.04 km^2^ grid level (Ψ). Two species occurrent model [52] for estimating species interaction factor (SIF) between gharial and mugger. Surveys were conducted in winter season of Chitwan National Park, Nepal. AIC is Akaike’s information criterion, ΔAIC is the difference in AIC value of the focal model and the best AIC model in the set, K is the number of model parameters and Deviance is −2 of the logarithm of the likelihood function evaluated at the maximum.

Species	Model	AIC	ΔAIC	w_*i*_	Model Likelihood	K	Deviance
**Single Species Single Season Model**							
Mugger	Ѱ(.), *p*(Survey)	550.62	0.00	1.00	1.00	6	538.62
	Ѱ(.), *p*(.)	569.29	18.67	0.00	0.00	2	565.29
Gharial	Ѱ(.), *p*(Survey)	633.88	0.00	0.94	1.00	6	621.88
	Ѱ(.), *p*(.)	639.35	5.47	0.06	0.06	2	635.35
Crocodile	Ѱ(.), *p*(Survey)	911.97	0.00	1.00	1.00	6	899.97
	Ѱ(.), *p*(.)	924.79	12.82	0.00	0.00	2	920.79
**Species Interaction Model**							
Gharial & Mugger Interaction	Ѱ_G_,Ѱ_M_,Φ(.),pG = rG,pM = rM,delta	1162.39	0.00	0.76	1.00	8	1146.39
	Ѱ_G_,Ѱ_M_,Φ = 1,*p*G = *r*G,*p*M = rM,delta	1164.70	2.31	0.24	0.32	7	1150.70

Ѱ_G_: Probability of gharial being present at location *i*, regardless of occupancy status of mugger; Ѱ_M_: Probability of mugger being present at location *i*, regardless of occupancy status of gharial; pG: Probability of detection of gharial, given mugger is absent; pM: Probability of detection of mugger, given gharial is absent; *r*G: Probability of detection of gharial, given mugger is present; rM:: Probability of detection of mugger, given gharial is present; Φ: Species interaction factor; delta: detection interactor factor.

**Fig 4:**
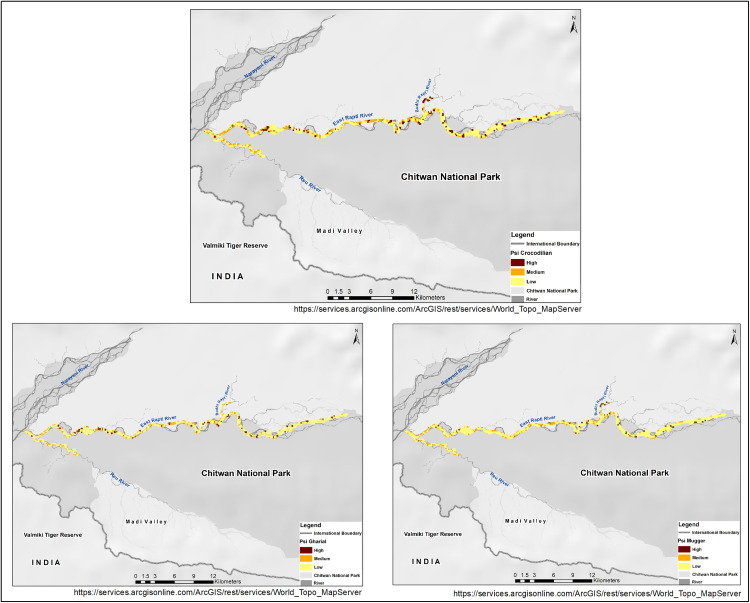
Survey grid cells (0.04 km^2^, coded in black for crocodilian sign detection) across the East Rapti River, Reu River, and Budhi Rapti River in Chitwan National Park, showing the probabilities of habitat use for crocodilians (4a:top), gharials (4b: down-right), and muggers (4c: down-left) [values are conditional occupancy probabilities estimates]. Map created using ArcGIS. Sources: Esri, USGS, NOAA (https://services.arcgisonline.com/ArcGIS/rest/services/World_Topo_Map/MapServer).

At the grid cell level (0.04 km²), we estimated the probability of detection (p̂, SE(p̂)) for crocodilians at between 0.03 (SE (0.02)) to 0.14 (SE (0.04)) per 200 m flight path searched, respectively. For individual species at the grid cell level (0.04 km²), we estimated the probability of detection (p̂, SE(p̂)) for gharials and muggers between 0.03 (SE (0.02)) to 0.08 (SE (0.04)) and between 0.09 (SE (0.04)) to 0.13 (SE (0.05)) per 200 m flight path searched, respectively. We calculated the site-level probability of habitat use $\left(\hat{\Psi }\;SE\left(\hat{\Psi }\right)\right)$ as 0.39 (SE (0.09), 69% increase from naïve estimate) for crocodilians in the Rapti River. For individual species, the site-level probability of habitat use $\left(\hat{\Psi }\;SE\left(\hat{\Psi }\right)\right)$ as 0.47 (SE: 0.20, 83% increase from naïve estimate, Fig 4) for gharials and 0.24 (SE: 0.09, 72% increase from naïve estimate, Fig 4) for muggers in the river stretch. By analyzing site-specific variation in habitat use, expressed as conditional occupancy probability, we found that both species intensively used certain habitats (categorized as riverbanks) more than others (in the river and/or floodplains) along the identified potential habitat (44.68 km^2^, Fig 4). We also estimated the grid-level probability of habitat use by both species combined (derived psiGM, ${\hat{\Psi }}_{\mathrm{GM}}$ ($SE\;{\hat{\Psi }}_{GM}$)) at 0.26 (SE: 0.13). Out of the total 44.68 km² of potential available habitat along the Rapti River and its tributaries, we estimated the total potential habitat area used at 17.42 km² (SE: 4.02 km^2^) for crocodilians, 21 km² (SE: 8.9 km^2^) for gharials, and 10.72 km² (SE: 4.02 km^2^) for muggers.

We evaluated spatial co-occurrence among crocodilians using SIF, which measures the degree of spatial association between species pairs. An SIF value greater than 1 indicates spatial aggregation, while a value equal to 1 suggests spatial independence or no interaction. For the crocodilian pair studied, we found strong statistical support for interaction between gharials and muggers, as indicated by a ΔAIC greater than 2.01 between models estimating the SIF and those assuming independence (SIF fixed at 1.0, Table 2). We estimated the SIF at 1.94 (SE = 0.56), which indicated significant spatial co-occurrence between the two species along the Rapti River rather than spatial avoidance. Additionally, the SIF differed meaningfully from 1.0, as the 95% confidence intervals did not include 1.0.

We observed variation in investment, including labor and field costs (expressed in US$, Table 3), between drone-based and traditional boat-based surveys. Drone-based surveys proved more efficient than boat-based surveys in terms of time and cost for covering the sampled areas. The cost of conducting a boat survey was significantly higher at ~ $21 per km, compared to just $0.61 per km for a drone-based survey, resulting in a substantial savings of approximately 97% when using drones instead of boats.

**Table 3:** Comparison of Survey Costs and Resource Allocation for Boat-Based and Drone-Based Surveys along a 73 km River Stretch in Chitwan National Park (44.68 km²). The costs presented include daily research expenses; overnight accommodation, and the costs of drones and boats are not considered. The boat survey followed the protocol outlined by Yadav et al., [22].

Types of Survey	Human Resources	Surveyed Distance	Surveyed Hours	Rate/Person/day	Person/day	Survey Days	Cost (in US$)	Total (in US$)	Cost Per km
**Boat**	Surveyor	72	133	15	4	21	1,292	1,507	20.93
	Data Supervisor			15	2	7	215		
**Drone**	Drone Pilot	694.2	13.3	38	1	5	192	431	0.61
	Drone Assistant			15	1	5	77		
	Image Processing			23	1	7	162		

## Discussion

This study demonstrates the use of drones for monitoring the largest population of threatened crocodilian species at scale in Nepal and contributes to the ecological understanding of these species in freshwater ecosystems in South Asia. Key findings includes (i) identification of the largest known population of critically endangered gharials (205 individuals) and vulnerable mugger (118 individuals) within 73 km of rectified drone imagery, (ii) Occupancy probabilities of 0.47 for gharials and 0.24 for muggers in their respective ranges, (iii) habitat occupied by gharials and muggers in riparian zones covered an area of 15.2 km^2^ and 7.7 km^2^, and (iv) evidence of co-occurrence between gharials and muggers along the Rapti River stretch.

This study complements ongoing ground-based surveys monitoring the gharial population [22,25] and serves as the first baseline assessment of crocodilian populations using aerial monitoring in the Rapti River, Chitwan National Park. The analysis, guided by a priori considerations, identified the largest observed population of critically endangered gharials from rectified drone images along the 73 km of river stretch. Comparisons with previous studies [22] suggest the population size may have been underestimated in the past, emphasizing the need for regular monitoring with drones. The basking season (winter, Table 1) [54], the timing of the survey (early morning or afternoon, between 11:00 to 16:00 ) [55], low turbidity [26], and the high resolution drone imageries (measured at 2 cm per pixel, present study) ensured the effectiveness of the drone platform for monitoring these crocodilians in riparian habitats and corroborate with previous studies [26].

The drone survey recorded a total of 205 gharials along a 73-km stretch of aquatic habitat in the Rapti River. This count is almost double the previous estimates reported by Yadav *et al.,* [22] (n = 96) and Poudyal *et al.,* [56] (n = 118) for the same river stretch. In 2024, just before the drone survey, CNP authorities conducted a traditional ground-based survey and counted a total of 152 individuals in the same area [30]. The discrepancies in gharial population counts between studies stem primarily from variations in survey protocols [26] and the periodic release of captive raised gharials into the wild [27]. Till 2025, a total of 1,305 gharials were released into the wild in the Rapti River. Additionally, factors such as survey timing [26,45], human disturbance levels [57], changes in river geomorphology [58], local weather conditions during the survey [26], and surveyor experience [22] can also contribute to variations in results. Nevertheless, this study, along with previous surveys in Bardia National Park [26], demonstrates the effectiveness of drone-based surveys [59,60] as a potentially less invasive method for monitoring free-ranging crocodilian populations in the wild [61].

Our drone survey protocols, incorporating advanced drones with vertical take-off and landing capabilities, enabled extended flight times at ~150 m altitude, allowing comprehensive coverage of the study area and rapid surveys of river stretches. This ensured adherence to the closure assumption, critical for accurate gharial population estimates. In their review on disturbance of drones on wildlife, Mulero-Pázmány et al. [28] provide suggestions to minimize disturbance such as flying at the highest possible altitude and using lawn-mower flight patterns which is what was done in this study. The 150 m agl flights still yielded high resolution imagery (2 cm per pixel) and ensured a disturbance-free survey protocol for monitoring sensitive crocodilian populations in the Rapti River. The present survey recorded no signs of alteration (such as changes in position or diving into the water) for the gharials and muggers along the drone’s path, indicating minimal to no disturbance (noise-free) during crocodilian monitoring. Additionally, the increased animal count compared to ground-based surveys indicates that crocodilians may not have responded to drone noise, which could otherwise lead to underestimation. It is important to note that their still could have been non-visual impact such as increased stress levels [62].

Thapa *et al.,* [26] emphasized using detection and non-detection data from drone images within an occupancy framework. By employing a grid-based approach with spatially replicated flight paths, we developed a detection history for gharial occurrences in the Rapti River. Our occupancy probabilities (mugger-gharial) ranged between 0.24 to 0.47 demonstrating the effectiveness of the occupancy framework when compared to naïve estimates (0.07 to 0.12). Yadav *et al.,* [22] estimated occupancy (~0.80) using 1 km spatial replicate segments from ground-based surveys. Our survey specific detection probability (*p* = 0.03–0.08) based on aerial monitoring. Factors such as appropriate season [63], survey timing [54], high resolution imageries [26,64], and the lack of behavioral responses from gharials (present study) likely contributed to this difference. However, we have not conducted a sensitivity analysis and/or using the co-variates affecting detection probabilities to confirm these findings. While methodological differences exist, including grid cell size and shape (0.04 km² square grids in our study Vs 1-km segments), the drone platform provides a robust opportunity to estimate detectability and habitat use probabilities without violating the closure assumption but at a scale.

As expected, gharial and mugger were found to co-occur spatially along the Rapti River. No previous studies exist to compare or discuss this co-occurrence between species in the study area. The availability of riparian habitats and soft substrate sand banks for basking and nesting provides an opportunity for both species to share overlapping territories without significant competition. Limited basking sites along the Rapti River, particularly during the winter, may have brought these species into closer proximity [25]. In the Chambal River, India, the habitat characteristics for basking and nesting varied between the two species [45]. During winter, factors such as slope variability, moisture content, sandbar presence, and riverbank features influenced site preferences for both species [45]. The primary resource requirements for gharials, which are predominantly piscivorous, and mugger crocodiles, which exhibit a broader dietary niche, differ significantly due to their distinct dietary preferences. Despite sharing similar habitat conditions in the Rapti River, differences in microhabitat preferences, such as nesting sites or water depth, are likely to reduce resource competition, facilitating their coexistence. Disturbances along the riverbank can also influence basking site selection for both species [45,54]. Such disturbances may further encourage co-occurrence in the core areas of national parks where these species were recorded. During the survey, ocular observations revealed that most individuals were basking during most of the day (11:00 to 18:00) suggesting no temporal segregation between the species, a finding consistent with observations in the Chambal River [45].

Drone surveys were more cost-effective (US$0.61 per km) than traditional boat surveys (US$ ~ 21 per km) in terms of field operations and laboratory expenses. Our findings align with previous studies on gharial monitoring in Bardia National Park, Nepal [26], and liana infestation in tropical forest in Malayasia [53]. The lower operating costs of drone surveys make them a more viable option for long-term monitoring, particularly in large or remote areas where boat access is challenging or expensive. This cost efficiency enables more frequent and widespread surveys, the collection of high-quality data [65], and the acquisition of ancillary habitat information (habitat coverage, river disturbance data etc.) at a lower cost [26].

Searching through a vast collection of drone images to identify crocodilian dorsal patterns can be as challenging as finding a needle in a haystack. To address this, artificial intelligence has been employed to train models that enhance the accuracy of identifying and re-identifying individuals in future surveys. For instance, Desai et al. [66] utilized drone imagery and deep learning techniques to identify free-ranging mugger crocodiles by analyzing their unique dorsal scute patterns. This approach along with online machine learning platforms [67] such as Picterra demonstrated high accuracy and efficiency, offering a promising non-invasive method for monitoring crocodilian populations. Use of occupancy modelling approach [35], employing repeated count data [68] based on drone platform ([26] and present study) may benefit in the robust estimation [69] of gharial [22] including mugger population along the long ribbon of aquatic habitat in CNP and elsewhere.

## Conclusion

This study validates drone-based surveys as a cost-effective, non-invasive approach for monitoring gharials and muggers in the Rapti River, advancing ecological understanding into these crocodilians species. High-resolution drone imagery (2 cm/pixel) and optimized protocols ensured minimal disturbance and comprehensive coverage of the 73 km river stretch.

Limitations include the absence of covariate-based occupancy analysis and methodological variations (e.g., grid cell size) that hinders comparisons with ground-based surveys. Future research should incorporate sensitivity analyses, standardize and update survey protocols, and AI-driven dorsal pattern recognition to enhance population monitoring. Long-term drone surveys, leveraging their low cost (US$0.61/km), and alongside studies on gharial-mugger population dynamics and co-occurrence dynamics, could strengthen conservation efforts across South Asian River systems.
